# Optical Biosensors Based on Photonic Crystals Supporting Bound States in the Continuum

**DOI:** 10.3390/ma11040526

**Published:** 2018-03-30

**Authors:** Silvia Romano, Annalisa Lamberti, Mariorosario Masullo, Erika Penzo, Stefano Cabrini, Ivo Rendina, Vito Mocella

**Affiliations:** 1 Institute for Microelectronics and Microsystems—Unit of Naples—National Council of Research, Via Pietro Castellino, 80131 Naples, Italy; ivo.rendina@cnr.it (I.R.); vito.mocella@cnr.it (V.M.); 2 Department of Molecular Medicine and Medical Biotechnology, University of Naples Federico II, Via S. Pansini 5, 80131 Napoli, Italy; annalisa.lamberti@unina.it; 3 Department of Movement Sciences and Wellbeing, University of Naples “Parthenope”, Via Medina 40, 80133 Napoli, Italy; mario.masullo@uniparthenope.it; 4 Lawrence Berkeley National Laboratory, Molecular Foundry Division, 67 Cyclotron Road, Berkeley, CA 94720, USA; epenzo@lbl.gov (E.P.); scabrini@lbl.gov (S.C.)

**Keywords:** photonic crystal, bound states in the continuum, biosensors, protein interaction

## Abstract

A novel optical label-free bio-sensing platform based on a new class of resonances supported in a photonic crystal metasurface is reported herein. Molecular binding is detected as a shift in the resonant wavelength of the bound states in the continuum of radiation modes. The new configuration is applied to the recognition of the interaction between protein p53 and its protein regulatory partner murine double minute 2 (MDM2). A detection limit of 66 nM for the protein p53 is found. The device provides an excellent interrogation stability and loss-free operation, requires minimal optical interrogation equipment and can be easily optimized to work in a wide wavelength range.

## 1. Introduction

Optical biosensors are widely-used devices that find many crucial applications such as health-care, pharmaceutical and food quality control, environmental analysis, security, and industrial and chemical process monitoring [1,2,3,4]. Due to their high specificity, sensitivity, small size, and low cost, optical biosensors offer great advantages over conventional detection techniques, allowing the direct and real-time identification of many biochemical molecules. In particular, many optical label-free biosensors measure the refractive index (RI) change induced by molecular interactions occurring over a sensitive surface. This variation is directly connected to the surface concentration or density of the analyte that—unlike fluorescence-based sensing configurations—can be detected with simple assay processes and without any alteration or labeling procedure perturbing the molecular interactions under study. A widespread class of label-free photonic biosensors exploits optical resonance effects. They experience a shift in the resonance wavelength (*λ*) in response to a change of the RI of the medium (generally a dielectric) surrounding the sensing area. This is the case of surface plasmon resonance (SPR) and localized SPR (LSPR) [5,6,7,8] configurations, which are typically characterized by high values of sensitivity (S = ΔRI/Δ*λ*). However, their main figure of merit (FOM), commonly defined as the ratio between the sensitivity and the resonant peak width, is strongly affected by the large plasmonic optical losses, inducing resonance broadening. In addition, the local heating and heat dissipation due to the metallic nanostructures result in possible biological material damages, and thus the possibility of near-field enhancement in loss-free dielectric material is of great interest [9].

Other promising configurations are based on photonic crystals (PhCs), slotted, ring, and disk nanocavities [10]. They are characterized by high sensitivity, ultra-compact size, and easy integrability with other optoelectronic circuits on-chip. However, some critical issues—mainly related to the complicated light coupling process in integrated nanostructures [11] and to the unpredictable fabrication imperfections and contaminations [12,13]—may affect their technological transfer from research laboratories to real-world applications.

Herein, we present the realization of a new label-free resonant optical biosensor overcoming many issues characterizing the previous configurations, and we successfully apply it to the study of a protein—protein specific interaction of great interest in cancer therapy. The device is based on a PhC nanostructure supporting a collective resonant effect due to presence of bound states in the continuum (BICs) [14,15,16,17,18,19,20,21]. BICs are special Fano resonances with a theoretical infinite lifetime, related to waves that remain localized even though they coexist with a continuous spectrum of electromagnetic radiations carrying energy away from the system [22]. BICs resonances are decoupled from the continuum by symmetry mismatch of the mode profile or modal interference. The finite extension of the structure and the random imperfections due to material fabrication and contaminations break the ideality of the system. Experimentally, it is possible to couple input light with these very narrow resonances having a high Q-factor, also because of the non-zero divergence of the input beam. As a consequence of the high Q-factor, they manifest an extremely large near-field intensity enhancement. Specifically, our numerical simulations conservatively predicted an amplification of a factor as large as six orders of magnitude with respect to the intensity of the incident beam [23]. At the boundaries, BICs can be considered as surface waves, since the optical field is localized at the interfaces between the PhC surface and the surrounding environment. In this way, structures supporting BICs are intrinsically distributed-cavities and can find direct applications in laser, filter, and sensor realization [24,25,26,27,28]. 

In particular, we apply this new BIC-based approach to the label-free recognition of a specific protein—protein interaction, detecting the association between the interacting domain of p53 and its protein regulatory partner murine double minute 2 (MDM2). The method can be extended to the study of the dissociation effect exerted by other molecules on the p53•MDM2 complex, even at the nM range, therefore being of great importance in the first approach towards the discovery of substances with pharmacological activity against cancer. Indeed, protein p53 is one of the most intensively studied tumor suppressor genes [29,30,31,32]. The epigenetic code resulting from its modifications can modulate its functions. Such modifications affect the regulation of all three steps required for p53 activation: (1) p53 stabilization, (2) DNA binding, and (3) transcriptional activation. The control of p53 stability is largely influenced by ubiquitination. This modification is mainly ruled by the oncoprotein MDM2, which is the principal cellular antagonist of p53. Generally, this kind of fundamental interaction is investigated by using different methods, including NMR technology [33,34] and fluorescence polarization [35] (all techniques requiring complex tagging/derivatization procedures), and more recently electrophoresis [36]. In contrast to these techniques, our approach potentially allows the real-time recognition and label-free identification of the proteins’ interaction at nanomolar concentration, since the very intense optical field at the surface and the minimal loss of our transparent device make these PhC structures extremely sensitive to the external environment perturbation. Moreover, in comparison with SPR-based techniques [37], the BIC-based configuration may be advantageously exploited in different wavelength ranges and within microfluidic architectures, avoiding the undesired presence of vortices in analyzed solutions due to the heating effect in plasmonic (metal) surfaces under light irradiation. In addition, in contrast to other planar PhC structures, and because of the large sensing area (1 mm^2^), our PhC-BIC sensor requires a simple optical setup and minimal alignment procedures, thus representing a promising platform for many sensing applications. This study represents a proof-of-principle experiment targeting the p53•MDM2 complex towards the screening of substances with pharmacological activity against cancer. Therefore, our results propose a new strategy to easily study bio-molecular interaction problems of general and challenging interest in biology and medicine. 

## 2. Materials and Methods

### 2.1. Design, Fabrication, and Optical Characterization

**Figure 1:**
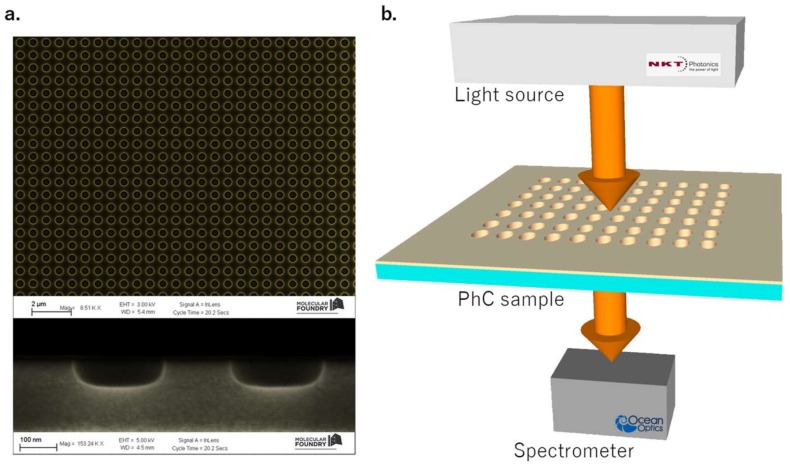
(**a**) Scanning electron microscopy image of the photonic crystal (PhC) sample. The design consists of air-cylindrical holes arranged in a square lattice (*a* = 521 nm, *r* = 130 nm, *h* = 78 nm). (**b**) Sketch of the experimental setup.

**Figure 2:**
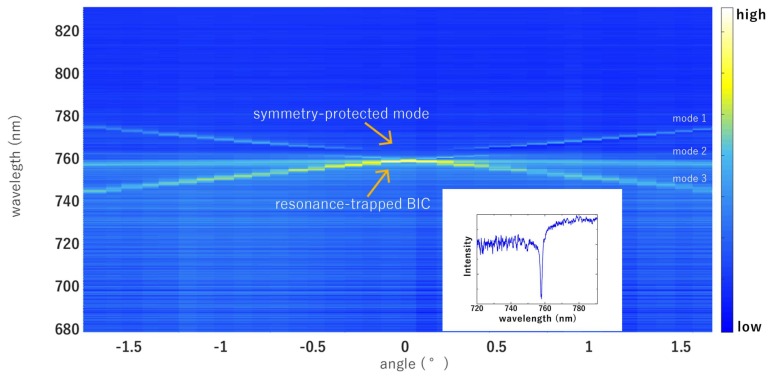
Dispersion band diagram along ΓX direction close to the normal incidence. The superior band is characterized by a vanishing linewidth towards θ = 0°. The first singly-degenerate mode 1 is a symmetry-protected bound state in the continuum (BIC). The inferior degenerate band (modes 2 and 3) instead shows avoided crossing resonances at Γ splitting, and is associated to a resonance-trapped BIC. The inset shows the transmitted spectrum at θ = 0°.

The PhC metasurface was realized on silicon nitride (Si_3_N_4_) deposited on a SiO_2_ substrate by means of plasma-enhanced chemical vapor deposition. The design was patterned on the deposited film by using electron beam lithography and was then transferred by means of coupled plasma etching process using CHF_3_ and O_2_. The lattice parameters were optimized in order to excite the BIC resonance at around 760 nm. Specifically, the lattice constant was *a* = 521 nm, the hole radius was *r* = 130 nm, and the hole depth was *d* = 78 nm (see Figure 1a). Preliminarily, before the functionalization process, the bare PhC membranes were optically characterized by means of a dedicated optical setup. Transmission spectra were acquired from the sample illuminated by a normally incident supercontinuum light (NKT Photonics) by using an Ocean Optics USB4000 spectrometer (Ocean Optics, Delray Beach, FL, USA) with a resolution of 0.25 nm. A sketch of the experimental setup is reported in Figure 1b. A computer-controlled rotational stage allowed angle-resolved measurement to be performed with a resolution of 0.01° [38]. The band diagram was reconstructed by changing the incident angle θ of the incoming beam (Figure 2). The BIC resonance appears as a dip in the transmittance spectrum (the inset of Figure 2 shows the transmission spectrum for θ = 0°). Two kinds of modes were found—one singly degenerate mode (mode 1), named symmetry-protected mode [39]; and two doubly degenerate modes (mode 2 and 3), named resonance-trapped BIC [24]. The first one is due only to the symmetry-mismatch with radiative fields. Its quality factor—independent of the holes diameter and generally from any variation of parameters that preserve the symmetry—decreased dramatically away from the normal incidence. The second ones are instead produced by destructive interference among modes and can occur at any wavevector because of resonance trapping [24]. In particular, these modes depend on the holes radius *r* and on the thickness *h* of the membrane, which are useful tuning parameters for increasing the Q-factor of the degenerate mode. At a singular radius *r_opt_* and *h_opt_*, the Q-factor becomes infinite but remains very high for values around these [24]. In contrast to symmetry-protected BICs, resonance-trapped BICs preserve a very high Q-factor within a broad angular range of incident excitation and this provides a good opportunity for real-world application. 

**Figure 3:**
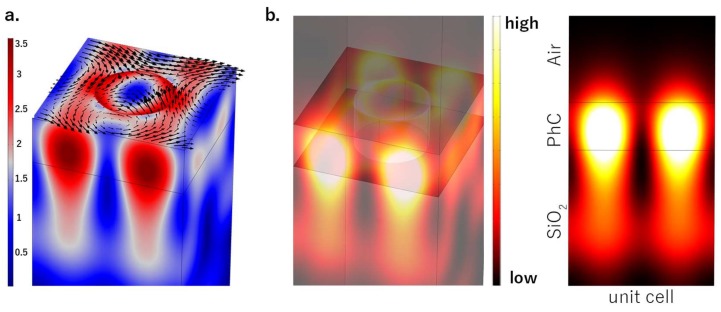
(**a**) BIC amplitude over the PhC with superimposed arrow maps of the electric field: as clearly visible, the electric field when a resonance trapped-BIC is coupled forms a lattice of vortices and antivortices that cannot couple to radiating waves since it is evanescent with no out-of-plane components of Poynting vector. (**b**) Intensity profile of the electric field and side view of one unit cell. The field is evanescent in both *z*-directions.

Numerical simulations of the PhC structure were carried out by using Comsol Multiphysics 5.2a (COMSOL INC., Stockholm, Sweden). Bloch periodic boundary conditions to surfaces along *x*- and *y*-directions were imposed to the unit cell, which represents the computational domain [23,40,41]. Far from the membrane, perfectly-matched-layer absorbing boundary conditions were imposed on top and bottom surface, normal to the *z*-direction. The size-step of the adapted mesh along *z* was 3 nm inside the PhC and became 20 nm outside. Figure 3a depicts the calculated mode of interest to our work—the resonance-trapped BIC—showing the electric field arrow map superimposed with the amplitude distribution. The BIC mode behaves as an evanescent surface wave that cannot couple to free-space modes (i.e., the electromagnetic field is confined in the near-field of the PhC surface). As is clearly visible, the field appears as a lattice of vortices and antivortices at the interface with the air. Figure 3b shows the intensity profile of the electric field and the side view. The electromagnetic field is mostly confined at the interface between the photonic crystal and the quartz substrate, but the field enhancement at the PhC/Air interface was supposedly high enough to provide a strong light–matter interaction over the surface.

### 2.2. Surface Modification

**Figure 4:**
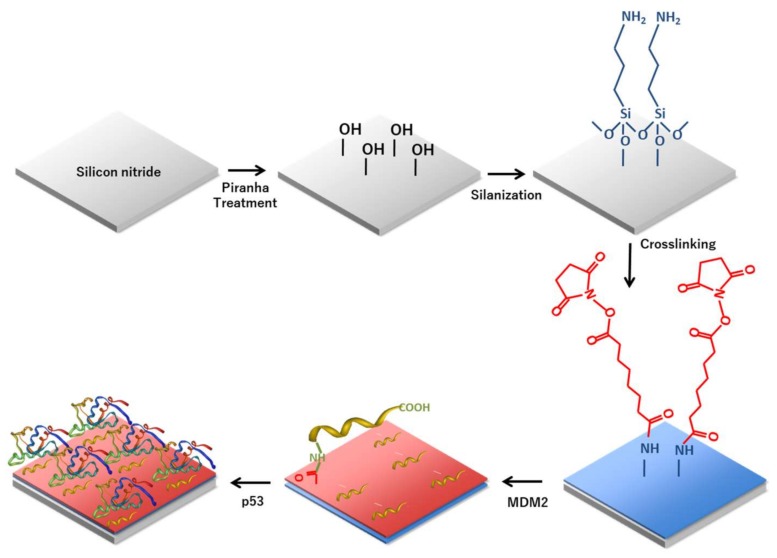
Functionalization approach utilized on silicon nitride surface to conjugate murine double minute 2 (MDM2) recombinant protein able to detect p53.

**Figure 5:**
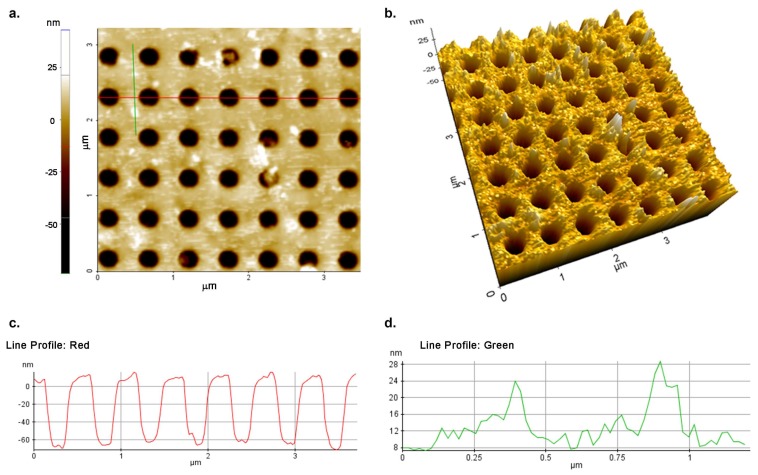
Atomic force microscopy (AFM) images and line profile of the functionalized PhC surface. Figure (**a**,**b**) reveal the presence of the p53•MDM2 complex over the PhC surface. The red profile (**c**) corresponds to the red line drawn over a row of holes, showing their cross-section, the green profile (**d**) to the green line drawn in an area containing proteins.

In order to functionalize the silicon nitride surfaces for the protein immobilization, the procedure described by Caballero et al. was used [42]. All the process steps are reported in Figure 4. Briefly, after a cleaning process carried out with organic solvents (hexane) and Milli-Q water (Millipore, Burlington, MA, USA), the silicon nitride sample was immersed in freshly prepared Piranha solution (H_2_SO_4_ 3.0 M, 30% H_2_O_2_) at 90 °C for 30 min. Then, sequential immersion in aqueous solutions of NaOH (0.5 M) for 20 min, HCl (0.1 M) for 10 min, and a final immersion in NaOH solution (0.5 M) for 10 min were performed to activate the surface. The samples were then extensively washed with HCl and water and dried in a nitrogen stream. The structure was then silanized in 5% (3-aminopropyl) triethoxysilane (APTES) (Appendix A) in dry ethanol by immersion at room temperature for 1 h. After silanization, the excess of unbound silanes was removed by rinsing the sample three times in ethanol for 2 min. The last step of the silanization process was curing on a heater at 100 °C for 10 min. The surface was then functionalized with BS^3^ (bis(sulfosuccinimidyl) suberate), an amino reactive crosslinker. To this aim, the substrate was covered with 10 mM BS^3^ in 20 mM HEPES buffer pH 7.5 for 5 h at 4 °C, rinsed twice with deionized water, and dried in a nitrogen stream [43]. Once modified with BS^3^, the protein MDM2 was immobilized on the chip surface by covering it with a 1.5 μM solution of the recombinant protein in HEPES 20 mM, pH 7.4 at 4 °C overnight. After the incubation, the substrates were washed first with HEPES buffer and then with 1× sodium phosphate buffer (PBS) pH 7.3. Furthermore, the chip surface was treated with 5% non-fat dried milk in PBS at room temperature for 1 h to reduce non-specific binding. In this way, the free reactive groups of the cross linker (BS^3^) were fully saturated and could not react with p53. Then, the sample was washed thoroughly with PBS 1× and dried in a nitrogen stream. Finally, the biofunctionalized PhC surface was incubated with a solution of recombinant protein p53 (1.5 μM in PBS) for 20 min at room temperature, extensively washed with PBS, and analyzed. Figure 5a,b shows the atomic force microscopy (AFM) images of the sample after protein–protein interaction and reveal the protein deposition over the surface. In particular, Figure 5c shows the profile of the holes corresponding to the area under the red line, whereas Figure 5d reveals the presence of the proteins in the area under the green line.

## 3. Results and Discussion

**Figure 6:**
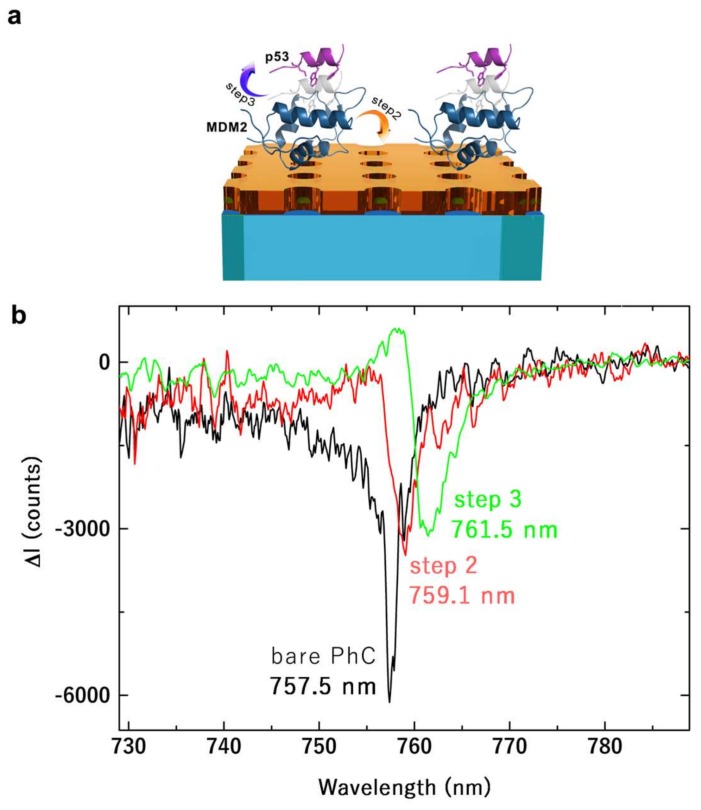
(**a**) Schematic layout of the two-dimensional square PhC with the MDM2 protein (step 2) dispersed over the whole sample surface and the subsequent interaction with p53 protein (step 3). (**b**) Measured transmitted spectra collected from the sensor device corresponding to the bare PhC (black curve), after the MDM2 deposition (red curve), after the interaction with p53 (green curve). Negative values of the intensity are due to background subtraction.

**Figure 7:**
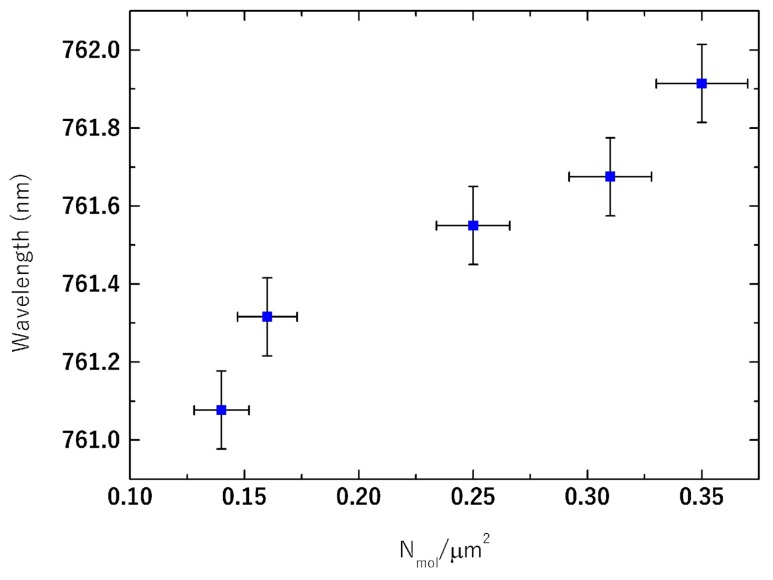
Resonance peak wavelength as a function of the p53•MDM2 complex surface density on the PhC.

Figure 6 shows the main result of this work. The reference spectrum is represented by the transmission signal of the bare PhC, before the functionalization, at θ = 0°. When the protein MDM2 linked over the PhC surface, a local modification in the refractive index occurred. This was transduced in a red shift in the resonance wavelength of 1.6 nm with respect to the reference spectrum. After the incubation with the recombinant protein p53, a further shift of 2.4 nm was detected. The shift was produced by the perturbation of the dielectric environment that induces a redistribution of the electromagnetic field, which was pushed towards the region of higher refractive index. As such, the resonant frequency also decreased and it produced a red-shift of the peak wavelength. In particular, this result is relevant because the monitoring was only limited to the few molecular binding events occurring on the surface of the PhC and not to a bulk sample as typically occurs in electrophoresis analyses. In other words, the sensitivity to the number of molecules involved in the process was much larger than what is typically achieved in conventional biochemical assays. To more accurately analyze the resonance-peak wavelength shift as a function of the concentration, we correlated it with the actual molecular surface density on the PhC estimated by AFM maps. We used the topographic maps to count molecular binding spots by considering the p53•MDM2 complex having a height in the range 10–20 nm. In particular, we estimated the surface density in AFM maps of 20 × 20 µm^2^. Then, we used a beam spot of approximately 50 µm diameter to independently measure the local resonance shift corresponding to the investigated area (depending on the surface density). The selected area was visually inspected with a CCD camera. With a p53 concentration of 1.5 μM and an incubation time of 20 min (see Materials and Methods section), the surface density was estimated to be on average about 0.24 N_mol_/µm^2^, with N_mol_ number of molecules. In Figure 7, we plotted the resonance shift as a function of the molecular surface density. Our results prove that the sensor is potentially capable of monitoring MDM2-p53 interaction with few-molecules sensitivity, which is our main outcome. 

In order to provide a direct comparison with the other sensing techniques, following the general discussion of PhC nanocavity sensitivity [10], let us consider the detection limit (C) of the PhC-based RI sensor. It can be demonstrated that C=ΔλminC1Δλ, where *C*_1_ is the experimentally detected concentration, Δ*λ* the corresponding shift, and $\Delta \lambda_{min}=\lambda_{0}/10$Q [10]. For our PhC-BIC sensor, $\Delta \lambda_{min}=$ 0.1 nm ($\lambda_{0}$ = 760 nm) and Q = 760, and this corresponds to a value of *C* = 66 nM, which is a remarkable result considering the other devices’ performances. It is worth mentioning that similar results can be achieved by exploiting SPR biosensors, which use a similar detection mechanism, but in that case protein p53 is biotinylated [37].

One of the main issues in real-world applications of PhC nanocavity devices is the impossibility of far-field light coupling. Generally, a conventional single-mode fiber is necessary, and the coupling losses can be high if the system is not perfectly aligned [11]. In contrast to these kinds of PhC sensors, our BIC-based sensor allows a simple and efficient far-field coupling that is not affected by fabrication imperfections and by the excitation angle definition. Thus, in order to verify the independence of the detection limit from the incidence angle of interrogation, angle-resolved measurements were performed. In Table 1, the detection limits determined for different incident angle—in a range of 2 degrees—are summarized. As can be seen, the fluctuation in C was within the uncertainty of the measurements (3 nM) and the same spectral shift regardless of the specific alignment could be detected because of the large Q-factor over a wide angle of incidence. This result is remarkable because it alleviates one of the major drawbacks of large-area mode resonance of PhC, i.e., the strong dependence on the alignment.

## 4. Conclusions

MDM2 and p53 have documented interactions that depend on several external biochemical constraints. Since a systematic study of the influence of these external factors on the proteins’ interaction would be highly advantageous in cancer research [29,30,31,32], a first crucial step in this direction is setting the ground for monitoring such an interaction in a label-free fashion, with a sensitivity level that might push further the sensing capability of the binding event recognition, and possibly with a non-invasive approach based on an optical read-out mechanism. This last, in particular, is highly challenging since it requires high figures of merit for achieving a large sensitivity. Our device exploits special kinds of Fano resonances to induce high optical field enhancement and confinement at the sensing surface. Based on this rationale, in this work, we have set an intermediate milestone by studying the first fundamental conditions under which MDM2-p53 interaction could be optically detected. We modeled, devised, and characterized an optical sensor based on a photonic crystal slab working at the BIC point of its parameters space. We determined the feasibility of the optical detection of the MDM2-p53 binding at a low level of surface molecular density. The interrogation scheme requires minimal far field optical equipment, and is extremely robust to optical misalignment. In addition, the sensing configuration is highly versatile, it can be optimized to work in a wide wavelength range, and its operation is free from interference of multiple modes. The device may have great potential for the realization of low-cost sensing platforms, eventually integrated in microfluidics, capable of operating with minimal sample volume and of detecting analytes at extremely low concentration levels. We achieved a detection limit of about 66 nM of p53. This sets the basis for our future work aimed at a comprehensive study of the dynamic range and kinetics of interaction as a function of the external biochemical factors acting on p53•MDM2 complex, even at the nM range, with the final aim of the low-cost investigation of molecules with pharmacological activity against cancer.

## Figures and Tables

**Table 1:** Detection limits determined for different incident angle of the beam. The fluctuation in C is within the uncertainty of the measurements (3 nM).

θ = −2°	θ = −1°	θ = 0°	θ = 1°	θ = 2°
69 nM	66 nM	66 nM	57 nM	69 nM

## Appendix A

### Chemicals and Reagents

(3-Aminopropyl)triethoxysilane (APTES), Bis(sulfosuccinimidyl) suberate (BS^3^), and H_2_SO_4_ were purchased from Sigma-Aldrich (St. Louis, MO, USA). HCl and hexane were purchased from Romil (Waterbeach, Cambridge CB25 9QT, UK). Absolute Ethanol, H_2_O_2_, and NaOH were purchased from Carlo Erba (Cornaredo, Italy), HEPES powder was purchased from Promega (Madison, WI, USA). The MDM2 and p53 protein fragments used in this work were heterologously expressed in *Escherichia coli* and characterized as previously reported [36,44]. Piranha solution was used according to the safety guidelines of University of Chicago Environmental Health and Safety (Chicago, IL, USA).
